# Impact on prostate cancer clinical presentation after non-screening policies at a tertiary-care medical center- a retrospective study

**DOI:** 10.1186/s12894-021-00784-w

**Published:** 2021-02-08

**Authors:** Tarek Ajami, Jaime Durruty, Claudia Mercader, Leonardo Rodriguez, Maria J. Ribal, Antonio Alcaraz, Antoni Vilaseca

**Affiliations:** 1 Urology Department, Hospital Clínic de Barcelona, C/ Villarroel, 170, 08036 Barcelona, Spain; 2 Urology Department, Hospital Fuerza Aérea de Chile, Santiago, Chile; 3 Pathology Department, Hospital Clínic de Barcelona, Barcelona, Spain

**Keywords:** Prostate cancer, Prostate specific antigen, Prostate cancer screening

## Abstract

**Background:**

In May 2012 the US Preventive Task Force issued a ‘D’ recommendation against routine PSA-based early detection of prostate cancer. This recommendation was implemented progressively in our health system. The aim of this study is to define its impact on prostate cancer staging at a tertiary care institution.

**Methods:**

A retrospective analysis was performed from 2012 until 2015 at a single center. We analyzed the total number of biopsies performed per year and the positive biopsy rate. For those patients with positive biopsies we recorded diagnostic PSA, clinical stage, ISUP grade group, nodal involvement and metastatic status at diagnosis.

**Results:**

A total of 1686 biopsies were analyzed. The positive biopsy rate increased from 25% in 2012 to 40% in 2015 (*p* < 0.05). No change in median PSA was noticed (*p* = 0.627). The biopsies detected higher ISUP grades (*p* = 0.000). In addition, newly diagnosed prostate cancer presented a higher clinical stage (*p* = 0.005), higher metastatic rates (*p* = 0.03) and a tendency to higher lymph node involvement although not statistically significant (*p* = 0.09).

**Conclusion:**

After the 2012 recommendation, patients presented a higher probability of a prostate cancer diagnosis, with a more adverse ISUP group, clinical stage and metastatic disease.

These results should be taken into consideration to implement a risk adapted strategy for prostate cancer screening.

## Background

Prostate cancer is the second most commonly diagnosed and the most prevalent cancer among males, with 358,989 deaths worldwide during 2018 [1]. Since the introduction of PSA-based prostate cancer screening in the late 1980s, the prostate cancer incidence increased considerably and reductions of up to 50% in mortality were reported [2–6]. However, the increased diagnosis also portends an increased overdiagnosis and overtreatment [7–9] with its related complications (mainly anxiety, sepsis, urinary incontinence and erectile dysfunction) [10–12]. The risk/benefit of prostate cancer screening became, and continues to be, a controversial topic.

In May 2012 the US Preventive Task Force (USPTF) issued a ‘D’ recommendation for routine PSA-based early detection of prostate cancer, stating that it should not be offered in the general U.S. population, regardless of age [13]. This recommendation was based on the results of two randomized trials willing to prove whether screening could reduce prostate cancer mortality. The “Prostate, Lung, Colorectal, and Ovarian (PLCO)” screening trial, in which 76,685 men between 55 and 74 years were randomized to either annual PSA screening and digital rectal examination for 6 years or ‘usual care’, showed no mortality advantage at 10 years follow up (RR of 1.11 [CI 0.75 to 1.70]) [14]. Longer follow up in the PLCO still fails to prove any benefit for screening (RR of 1.04 [95% CI 0.87–1.24]) at 15 years [15]. The European trial (ERSPC) randomized 182,160 men between the ages of 50 and 74 years and found a statistically significant 21% reduction in prostate cancer mortality in men between 55 and 69 years (RR of 0.79; [CI 0.68–0.91; *p* = 0.00]) at 11 years follow up [16]. The aim of this study is to analyze the impact of the 2012 recommendation at our institution in terms of prostate cancer diagnosis and clinical staging.

## Methods

After obtaining the institutional ethics committee’s approval, we conducted a retrospective review of all patients who underwent prostate needle biopsies (PNB) at a single tertiary-care institution between January 2012 and December 2015. Patients were excluded from the analysis if they had been previously diagnosed with prostate cancer or had prostatic intraepithelial neoplasia (PIN) or atypical small acinar proliferation (ASAP) in the absence of any prostatic adenocarcinoma. We analyzed the total number of biopsies performed per year and the positive biopsy rate. For those patients with positive biopsies we recorded diagnostic PSA, digital rectal examination (DRE), ISUP grade group, nodal involvement and metastatic status. The Chi square test of independence was used to compare positive biopsy rates, Mann–Whitney U test was used to compare prebiopsy PSA and Chi square Mantel–Haenszel test (linear by linear) for temporal tendency in the rest of the variables. Statistical significance was set at a *p* value < 0.05. Analysis was performed with SPSS 23.0 version.

## Results

During the period studied, 1686 prostatic needle biopsies were performed. An overall 45% reduction was observed in the number of biopsies performed between the first and last year studied. Table 1 shows the total number of biopsies and the positivity rate per year. The percentage of positive biopsies were 25% in 2012, 24% in 2013, 38% in 2014 and 40% in 2015, representing a significant increase (*p* < 0.0001).

**Table 1:** Population demographics and rate of positive biopsies

	2012	2013	2014	2015
Mean Age (SD)	69.1 (7.8)	68.7 (8.4)	69.1 (9.1)	71 (8.9)
Median PSA	9	9.5	8.3	8.3
Prior MRI (%)	10 (6.5)	29 (25)	43 (43)	54(40)
Positive biopsies (n)	152	118	100	135
Negative biopsies (n)	446	378	163	196
Total number of biopsies (n)	598	496	263	329
Positive biopsy rate (%)	25	24	38	40

The clinical presentation based on digital rectal examination (DRE), ISUP grade and distant metastasis were significantly worse in the later years studied. The lymph node involvement showed a non-statistically significant increase and the PSA value at diagnosis did not show any difference throughout the study period.

**Fig. 1:**
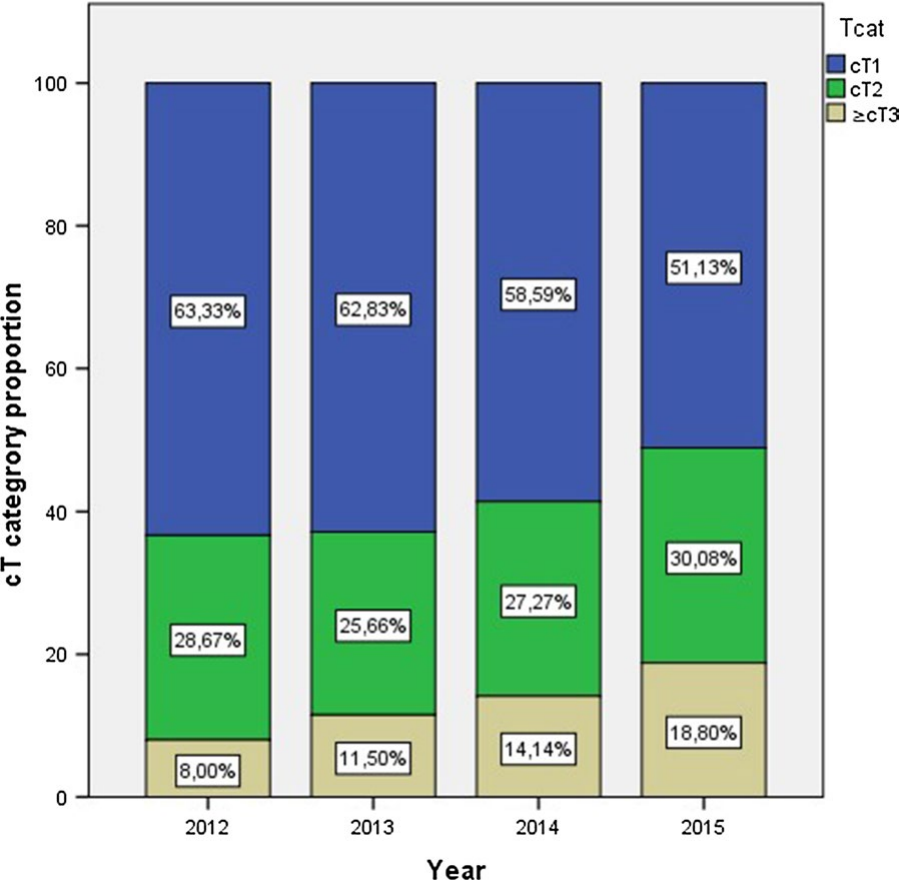
Clinical stage (DRE)/year

**Fig. 2:**
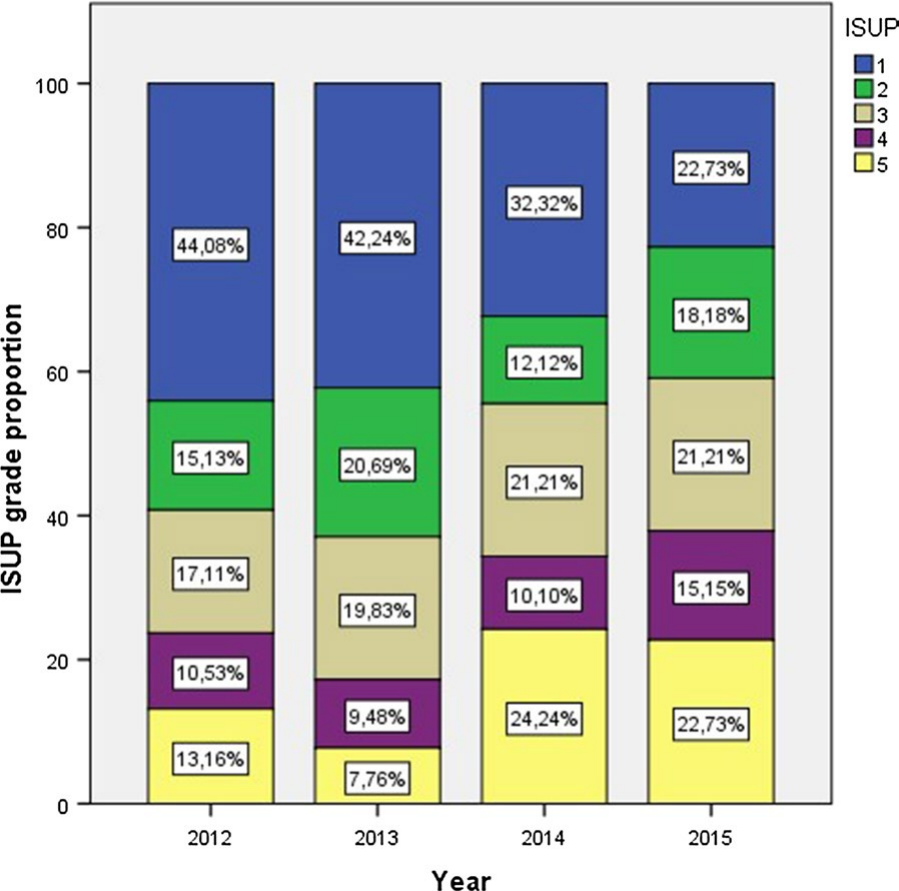
ISUP distribution/year

The proportion of clinical stage T1 and ≥ T3 were 63.3% and 8.0% in 2012. 62.8% and 11.5% in 2013. 58.6% and 14.1% in 2014 51.6% and 18.8% in 2015 respectively (*p* = 0.005) as shown in Fig. 1. Figure 2 summarizes the ISUP distribution per year. A significantly higher ISUP grade was seen in the linear temporal trend test (*p* = < 0.05).

**Fig. 3:**
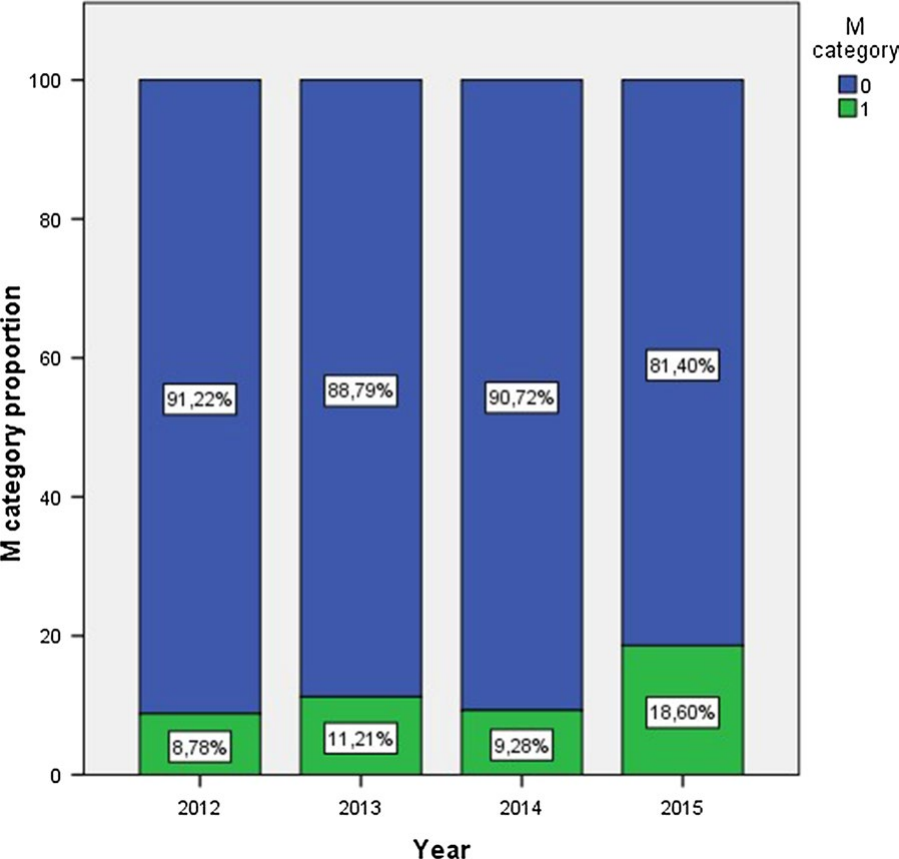
Distant metastasis at diagnosis/year

Significant differences were observed in distant metastasis at diagnosis (linear by linear chi square test *p* = 0.024) with a proportion of 8.8%. 11.2%. 9.3% and 18.6% each year (Fig. 3). An increasing trend in lymph node involvement was observed with a proportion of 10.8%. 13.1%. 11.3% and 18.6% yearly. However, these differences did not meet conventional levels of statistical significance (*p* = 0.09).

**Fig. 4:**
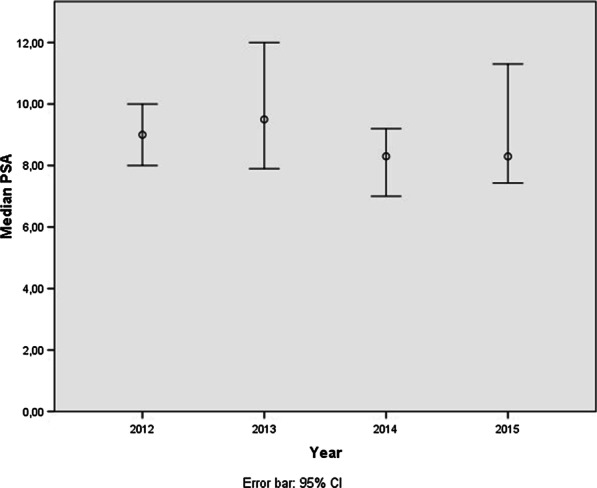
Annual median PSA

Surprisingly, the median PSA was 9.0 ng/dl (ICR 6.1–14.3) for 2012; 9.5 ng/dl (ICR 6.3–23) for 2013; 8.3 ng/dl (ICR 5.9–17) for 2014 and 8.3 ng/dl (ICR 6.01–21) for 2015 (Fig. 4). This difference in PSA values did not show a statistically significant difference (*p* = 0.627).

## Discussion

After the recommendation against massive prostate cancer screening by PSA, many studies have shown an increase in the diagnosis of high grade, locally advanced and metastatic prostate cancer [22–24]. Our results showed a significant impact of the screening policies, with a 45% decrease in the total number of biopsies performed per year and a significant increase in positive biopsy rates between 2012 and 2015. Our results confirm the findings of other groups that also found a significant decrease in the median number of biopsies [25, 26] and a 29% increase in positive biopsy rate [26]. Contrary to what we expected, our study was not able to show a significant increase in PSA at initial presentation, which was a constant in our examination of the literature [23, 28–30]. Although we are not able to provide a definite explanation for this finding, we believe it might occur because of the different derivation criteria of the associated centers, different levels of compliance with the indication not to perform PSA screening and the rising use of prebiopsy MRI in the studied period.

We observed a significant increase in local tumoral aggressiveness, mostly because of an increase in the clinical staging cT2-4, and an increase in ISUP 4 and 5. In their study, Banjeri et al., reported similar findings, with higher clinical stage (cT2b, *p* = 0.003; cT2c-3a, *p* = 0.027) and with D’Amico high risk scores (*p* = 0.036) after the USPTF recommendation [29]. We analyzed histological aggressiveness using the ISUP grading system. Several authors reported their results using the Gleason score and found a significant increase in grade [26, 30, 31, 41]. This increase in the diagnostic Gleason score has also been confirmed in the final pathology for radical prostatectomy [32].

We identified a significant increase in metastatic prostate cancer at the time of initial diagnosis, which, in our opinion, is the most important negative consequence of the implementation of non-screening recommendations. Our data supports the results described by other authors showing an increased incidence of metastatic prostate cancer at time of diagnosis. Bernstein et al., analyzing the SEER database, reported that in men ≥ 75 years old, the diagnosis of distant metastases increased in 2012 compared with 2011 (IR 1.13, 95% CI 1.02–1.24, *p* < 0.05) [33]. Using the same database, Hu et al., confirmed this increase between 2010 and 2013 both in men < 75 years (2.7%; 95%CI, 2.5%-2.9% vs. 4.0%; 95% CI 3.8–4.2%) and > 75 years (6.6%; 95% CI 6.2–7.0% vs. 12.0%; 95% CI 11.2–12.7%) [34]. In a population-based data review from 18 SEER registries, Dalela et al., noted that the incidence of metastatic prostate cancer increased significantly between the years 2009 and 2013 at a rate of 3.1% per year (*p* < 0.05) [35]. Interestingly, Weiner et al., found that the increase in the annual incidence of metastatic prostate cancer was higher among men aged 55–69 years [36]. This is especially worrisome, because this is the group most likely to benefit from definitive treatment. We failed to confirm a significant increase in pelvic lymph node metastasis, although we observed a rising trend, similar to the results obtained by Blair et al. It is worth noting that these results contrast with those reported by Bernstein et al., who, by analyzing the SEER database, showed a significant increase in pelvic lymph node metastasis between 2004 and 2014 (from 54.1 to 79.5 per million men (IR 1.47, 95% CI 1.33–1.62, *p* < 0.01) [33].

Updated results from the ERSPC trial confirm the risk reduction of developing metastasis (HR 0.70; 95% CI 0.60–0.82; *p* = 0.001) and PCa mortality, with a lower number of men needing to be invited for screening and diagnosis to prevent prostate cancer death (570 and 18 respectively) [37, 38]. It is important to mention that, in a predictive model, discontinuation of screening eliminates all overdiagnoses, but it doubles metastatic cases at presentation and increases prostate cancer deaths by 13–20% [39]. Different models for PCa screening have been developed, taking into account PSA and age, as well as other secondary tests, such as markers or imaging tests, prior to biopsy [40].

Although our study provides important information about the impact of the 2012 recommendation on PSA screening in our population, we are aware of its limitations. In addition to its retrospective design and inherent biases, as a tertiary center with different associated centers we do not know the full level of penetration of the recommendation and the exact time of adoption in primary health centers. In addition, there could be a selection bias as the patients included in the study were not segregated as to whether they are on a screening or non-screening protocol for PNB.

## Conclusions

After the recommendation against PSA screening, the diagnostic profile of prostate cancer has changed in our tertiary care institution, with prostate cancer being diagnosed at a higher clinical stage, with increased histological aggressiveness and increased risk of metastatic disease. Such findings should favor a PSA- based screening policy for early detection of PCa. Advancing in this direction, different urological societies are working to implement prostate cancer screening for the general population.

## Data Availability

The data supporting the conclusions used and/or analyzed in this study are available from the corresponding author by request.

## Abbreviations

PSA: prostate specific antigen
DRE: digital rectal exam
PIN: prostate intraepithelial neoplasia
ASAP: atypical small acinar proliferation
PNB: prostate needle biopsy
